# A study on biomedical researchers’ perspectives on public engagement in Southeast Asia

**DOI:** 10.12688/wellcomeopenres.19040.2

**Published:** 2023-09-08

**Authors:** Han Tran Dong Thai, Huong Van Thuy Qui, Thanh Vu Duy, Jaom Fisher, Mary Chambers

**Affiliations:** 1 Public Engagement Department, Centre for Tropical Medicine, Oxford University Clinical Research Unit, HCMC, 700000, Vietnam; 2 Nuffield Department of Medicine, Tropical Medicine and Global Health, Oxford University, Oxford, OX3 7LG, UK

**Keywords:** Public engagement, science communication, biomedical research, Southeast Asia, Vietnam, Nepal, Indonesia

## Abstract

**Introduction**: Public engagement is increasingly promoted in the scientific community. Although there are studies about researchers’ perspectives on public engagement, these are predominantly from Global North settings and there is little data from the context of Southeast Asia. The Oxford University Clinical Research Unit (OUCRU) is a clinical and public health research programme with sites in Vietnam, Nepal and Indonesia. There is a dedicated public engagement team, and it is recognised as an important part of the research process.

**Methods**: Through this study we explored the views and needs of local researchers with regards to practicing public engagement. We obtained opinions of 70 researchers through an online survey with both open-ended and closed-ended questions.

**Results**: Most researchers perceived public engagement as improving public science literacy, rather than supporting public participation in science and research. While the participants largely see public engagement as a necessary practice, they experienced four main barriers to taking part in public engagement: time, lack of capacity, lack of support and personal perceptions. Most participants indicated they had somewhat to low confidence to communicate about science to the public. Experience, skill and knowledge, and personal preference emerged as factors that influence their perceived confidence for science communication. In our analysis, experience appeared to be the main factor contributing to researchers' high confidence.

**Recommendations**: We recommended to support researchers by not only providing them with training for skills and knowledge, but also with opportunities to conduct public engagement, and a range of methods to suit their personal styles of communicating. It is also evident that more support is needed to build an enabling institutional environment that gives researchers professional recognition for their engagement work. This study, while modest in its scope, has informed our approach to supporting researcher-led engagement, and may guide other institutes wishing to improve this.

## Introduction

With the advancement in science and technology, and the current critical global issues such as the COVID-19 pandemic and climate change, it is essential to foster a strong relationship between science and society. Engaging with the public has become increasingly important in strategies of research institutions to ensure their research is socially relevant, and to build trust with the public. Public engagement describes the "myriad of ways in which the activity and benefits of higher education and research can be shared with the public. Engagement is by definition a two-way process, involving interaction and listening, with the goal of generating mutual benefit” ( National Coordinating Centre for Public Engagement, 2020).

Researchers are being called upon to engage in dialogues about the societal impact of their work with community members and the wider public ( Bodmer, 1985; Leshner, 2003; Mayor, 1999). Several large-scale initiatives have been established to support public engagement with science, including the Horizon2020 Funding Scheme by the European Union, the National Coordinating Centre for Public Engagement (NCCPE) in the United Kingdom, and Center for Public Engagement with Science and Technology created by the American Association for the Advancement of Science (AAAS).

Over the past four decades, The Wellcome Trust (United Kingdom) has invested in major research programmes in Africa and Asia, and intentionally supported engagement with the public to create people-centred health research. The Oxford University Clinical Research Unit (OUCRU), a large-scale clinical and public health research unit with site offices in Viet Nam, Indonesia, and Nepal, and is a Wellcome Trust Africa and Asia Programme. OUCRU’s Public and Community Engagement department (PCE) and engagement activities have been largely supported by Wellcome Trust core funding [106680/B/14/A], as well as through Wellcome International Engagement Awards and through research funding which has included engagement activities. 

There have been several studies about the motivation and barriers for scientists to communicate their science to the public. These factors commonly include time constraints, lack of funding, lack of skills, lack of opportunities, lack of enabling environments, etc. ( Cerrato *et al.*, 2018; Ho *et al.*, 2020; Iqbal & Kar, 2022; Poliakoff & Webb, 2007; Riley *et al.*, 2022; Woitowich *et al.*, 2022). However, the environment for public engagement in the context of Southeast Asia is still largely unexplored. There is little research on the public’s attitude towards science and how they view themselves having a role in scientific research. Although there is evidence in the Global North countries, such as Australia, the United Kingdom and the United States, that the public generally hold high credibility for science and scientists ( Ipsos, 2011; Pew Research Center, 2022; Wellcome, 2016), there are few surveys available describing the situation in the Southeast Asia. One global survey ( Wellcome Global Monitor, 2018) found the public in this region has low understanding of science and mostly low to medium trust in scientists, with a sizeable portion of people had no opinion about trust in scientists. In addition, the Southeast Asian cultures may differ from those in the Global North in that generally science is seen as external to local culture, and public participation in decision making varies considerably. For example, public participation may be common in the Philippines, whereas it is rare in Vietnam, Laos and Cambodia, and for many countries the role of the public in policy and public agenda is undefined ( Hofman, 1998; Lin Heng, 2002). A previous study comparing Taiwanese and German scientists’ orientation towards the media provides relevant insights about the practice of communicating science with the public in a modernizing Asian country. The study reported that scientists in Taiwan had lower priority for interactions with the media and were less likely to adapt to the journalistic style and relate their research to the social context and experience (Lo & Peters, 2015). In Southeast Asia, there is still little or no data describing the perspectives and attitudes of researchers about communicating and engaging the public with science.

In 2019 the OUCRU PCE department launched an online survey to explore how researchers in OUCRU units in Vietnam, Nepal and Indonesia and other partner research institutes perceive public engagement and the motivations and barriers that they face. The survey aimed to capture the views and needs of researchers who work at OUCRU or have been involved with OUCRU public engagement activities. This survey aimed to:

1. Assess understanding and attitude of researchers towards public engagement with science.
2. Explore researchers’ capacity and identify the barriers to public engagement for researchers.
3. Understand researchers’ specific needs (for training, funding, etc.) and develop recommendations for the OUCRU PCE team to support researchers.

This survey, while modest in its scope, enables us to start to understand the barriers and enablers to public engagement with science, and has informed our approach to better supporting researcher-led engagement. These findings are likely to represent the situation for researchers in other Southeast Asian institutes and may serve as a helpful reference for any initiatives in the region that aim to support researchers to implement public engagement in their work. These findings will also contribute to the global understanding of public engagement practice in different contexts.

## Methods

We used an operational research approach using a cross-sectional survey to collect data to inform our engagement programme. 

### Survey scope

A survey was designed using Google Forms. The survey included six closed-ended questions (multiple choice and Likert scale) and five open-ended questions. The questions covered three topics, namely: researchers’ perception about public engagement; researchers’ capacity and barriers to communicating science with the public; researchers’ need for support to communicate science with the public ( Table 1). This survey was developed based on questions from two surveys previously published by the Wellcome Trust ( Luck, 2016; Wellcome, 2016). The survey was in Vietnamese and English language.

**Table 1.** Survey questions.

	Questions	Type of questions
**Researchers’ perception** ** about public engagement**	(1) In your own words what do you think Public Engagement is?	Open-ended question
	(2a) How do you rate the necessity of communicating science to the public (non-science audiences)? (2b) Please justify your rating.	Options: Highly needed, Needed, Neutral, Unnecessary, Highly unnecessary and Don’t know. Open-ended question
	(3a) Are there benefits that researchers will gain for communicating science to the public? (3b) Please explain your answer.	Options: Yes/ No Open-ended question
**Researchers’ capacity and** ** barriers to communicating** ** science with the public**	(4) What are barriers to you doing more public engagement? You may list the 3 most significant.	Open-ended question
	(5a) What do you rate for your confidence in communicating science to the public? (5b) Please justify your rating.	Rating: 1 to 10 Open-ended question
	(6a) Have you ever taken part in a science communication activity with the public? (6b) If yes, how many times?	Options: Yes/ No Options: 1-3 times, 4-10 times, >10 times
**Researchers’ need for** ** support to communicate** ** science with the public**	(7) What are your suggestions to strengthen your science communication skills to the public?	Multiple choice with an option for adding their own suggestions.

### Data collection

The link to the survey was emailed to 320 contacts in total, which include (1) researchers (research assistants, PhD students, post-docs, etc.) in OUCRU in Vietnam, Indonesia and Nepal. There were 273 people on the OUCRU academic email group. (2) 33 Vietnamese science lecturers, professionals and researchers from local universities and science institutions whose contacts had been kept, with consent, following their participation in public engagement activities previously organised by OUCRU, and (3) 14 research institutions in Vietnam in the field of health, biomedical science, biology and biotechnology. Eight of these institutes were known because of previous research partnerships with OUCRU and six were identified through their institutional websites. The invitation informed people that the survey would take approximately 15 minutes to complete and that their identity and the information they provided would be kept confidential. The survey was initially opened for 15 days and was extended to 33 days to allow more responses (from 31 Jul to 1 Sep 2019).

### Data analysis

Following closure of the survey, the data was automatically mapped in Microsoft Excel, a feature of Google Forms, and cleaned for duplication and errors. Microsoft Excel tools were used to analyse and visualise the data. The English and Vietnamese data were combined in a single database for analysis by bi-lingual authors. For the close-ended questions, we conducted descriptive analysis for an overview of characteristics of the respondents and to find the most common perspectives and experiences shared by respondents. The open-ended questions were thematically analysed using inductive coding, in which themes emerge from researcher’s interpretation of raw textual data. The coding scheme was established by the first author after reading the data set. The second author then used the developed coding scheme to analyse the data separately. Subsequently, the two coders had final discussion to review and resolve on differences. The differences were minimal, so there was no change in the initial coding scheme. We also discussed with the other authors to agree with the final analysis and interpretation of the data.

### Ethical considerations

The study did not require ethical approval (per the Oxford Tropical Research Ethics Committee guidelines) as the primary audience were internal OUCRU staff, the objectives were to inform PCE department strategy, and the surveys were anonymous. At the start of the survey, the objectives of the survey were clearly stated, and the participants were informed that the anonymised, aggregated results may be published in the future. Participation in the survey was entirely voluntary with fields seeking personal information being non-mandatory and therefore completion of the survey was taken as consent to participate. Participation and distribution by representatives from the 14 external research institutes was a demonstration of institutional approval and consent.

## Results

70 people responded to the survey email invitation (response rate: 21.9%, out of 320 contacts emailed). The participants were mostly from OUCRU-Vietnam (56, 80%), with a minority from other institutions in Vietnam (7; 10%). The remaining participants were researchers of OUCRU Indonesia (5; 7.1%) and OUCRU Nepal (2; 2.9%). Of the 70 people responding at least five were researchers from outside of Southeast Asia, but not everyone included identification details so there may have been more.

**1. Researcher’s perceptions about public engagement**

***a) Public engagement is commonly perceived as improving public literacy and understanding of science***

When asked to define public engagement (question 1), the participants’ responses generally mapped onto one of two views: either public engagement was seen as improving public literacy and understanding of science; or public engagement was public participation in science and research. Some participants provided definitions that include both of these views. Nevertheless, most of the participants (67/70; 95.7%) indicated in their response that public engagement is improving public literacy and understanding of science. Fewer participants (22/70; 31.4%) recognised “engagement as a two-way process, involving interaction and listening, with the goal of generating mutual benefit” ( National Coordinating Centre for Public Engagement, 2020) in their responses. Table 2 and Table 3 show a breakdown of the different groups of perspectives constituting the two views about public engagement. A few of the participants in the survey (5/70; 7.1%) appeared to be unsure of the concept of public engagement as they simply stated public engagement to be ‘communicating science to the non-science public’ without additional elaboration.

**Table 2.** Participants view public engagement as improving public literacy and understanding of science (Question 1).

PUBLIC LITERACY AND UNDERSTANDING OF SCIENCE (TOTAL 95.7%)			
Translate science to lay audience to **make** ** science accessible** to all	**Sharing knowledge** to increase literacy or change behaviour	For public to understand science and research better to **increase** ** interest, support or appreciation**	**Maximize application** ** and impact** of science and research
32.9 %	28.6%	25.7%	8.5%

**Table 3.** Participants view public engagement as public participation in science and research (Question 1).

PUBLIC PARTICIPATION IN SCIENCE AND RESEARCH (TOTAL 31.4%)		
**2-way communication** with public to understand public opinions	For **public to be involved** ** or participate** in scientific research process	Create **transparency** in the way research is conducted
12.9 %	12.9%	5.7%

***b) Most participants thought that science communication with the public is necessary and greatly benefits research***

**Figure 1.**
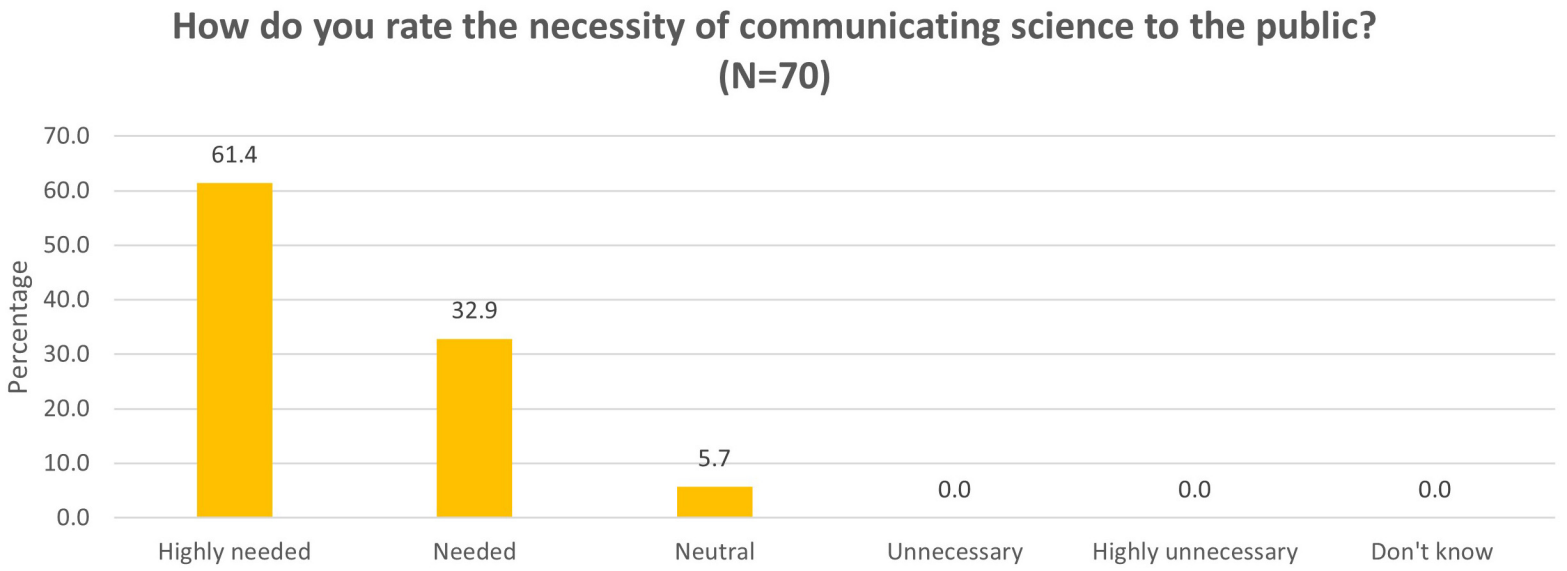
Participants rated the necessity of communicating science to the public (Question 2a).

Overall, the majority of the participants thought it is necessary to communicate science to the public (94.3%, 66/70). None of the participants thought this practice was unnecessary, yet there were a small portion of participants (5.7%, 4/70) who took a neutral stand ( Figure 1). Similarly, most of participants agreed that there are benefits from communicating science to the public (97.1%, 68/70) while there were 2 participants who indicated that science communication had no benefit (2.9%, 2/70). The participants recognised that communicating science to the public held more benefits for the research than benefits for the public or the researchers themselves. Participants were asked to explain why they think communicating science to the public is beneficial. The identified benefits of science communication are summarised in Table 4. The most common views include: public engagement benefits research through increased public support (21, 30%), to help understand public perceptions (21, 30%) and to ensure research is relevant (17, 24%). The next most common view was that public engagement can benefit the public by increasing public understanding of research and knowledge (15, 21%).

**Table 4.** Benefits of science communication perceived by participants (Questions 3a and 3b).

Are there benefits that researchers will gain for communicating science to the public?		Count	Percentage (N=70)
**Yes, there are benefits.**		**68**	**97.1**
**Benefits for** **research**	Gain public perspectives (learn from public knowledge, ideas)	21	30.0
	Public support (money, participation)	21	30.0
	Ensure and direct research to be real-life relevant	17	24.3
	Improve research work	10	14.3
	Increase trust in science and research	3	4.3
**Benefits for the** **public**	Increase public understanding of research and knowledge	15	21.4
	Apply science to improve life quality	6	8.6
	Inspired about science	1	1.4
**Benefits for the** **researcher**	Skills	7	10.0
	Motivations and inspiration	5	7.1
	Joy	1	1.4
**Uncertain**	Uncertain reasons	2	2.9
**No, there’s no benefit.**		**2**	**2.9**
	I am not sure.	1	1.4
	It’s time-consuming without bringing any profit for the research team.	1	1.4

Though most participants felt communicating science with the public is necessary, a small portion of the participants felt neutral about this. Table 5 shows the quotes from these neutral participants and a summary of their responses. Primarily concerns about the public, the complexity of science work and a need for support that leads the participants to take a more neutral stand towards science communication with the public. These negative views are helpful to understand the barriers for scientists to do more engagement work.

**Table 5.** Participants responses for feeling neutral about the need to communicate science with the public (Question 2b).

Quotes	Issue
“Research fields are too specialised, so only need to communicate to public pieces of information that they can understand…”	Complexity of the subject
“…with the current level of public’ literacy, we should consider if public wants to know about science and research.”	Concern about public (interest, literacy level)
“…researchers are too busy… requires money and a team with passion for communicating science.”	Need of support
“…General public is not familiar with the field… General public often do what researchers want them to do.”	Concern about public (interest, literacy level)

**2. Reported barriers towards public engagement and researchers need of support**

***a) Barriers to be involved in public engagement***

Participants were asked to list the barriers for them to do public engagement in an open-ended question (question 4). The responses were coded into 4 common themes as shown in Figure 2a, Figure 2b. For the three barriers (lack of support, lack of capacity and personal perceptions), responses were put into sub-groups to highlight specific needs for having public engagement capacity, specific support to do public engagement and specific perception-related barriers ( Table 6, Table 7 & Table 8). The most commonly reported barrier was the lack of capacity (55/70, 78.6%; Figure 2a). Most participants emphasised the need to have skills, ideas and knowledge of the different ways to communicate science to lay audiences; and the ability to use appropriate language when communicating science to lay audiences. A smaller portion of participants attributed public speaking skill as a part of public engagement capacity. Participants also thought understanding the concept of public engagement, and understanding public audiences are needed to be capable of doing public engagement.

**Figure 2a.**
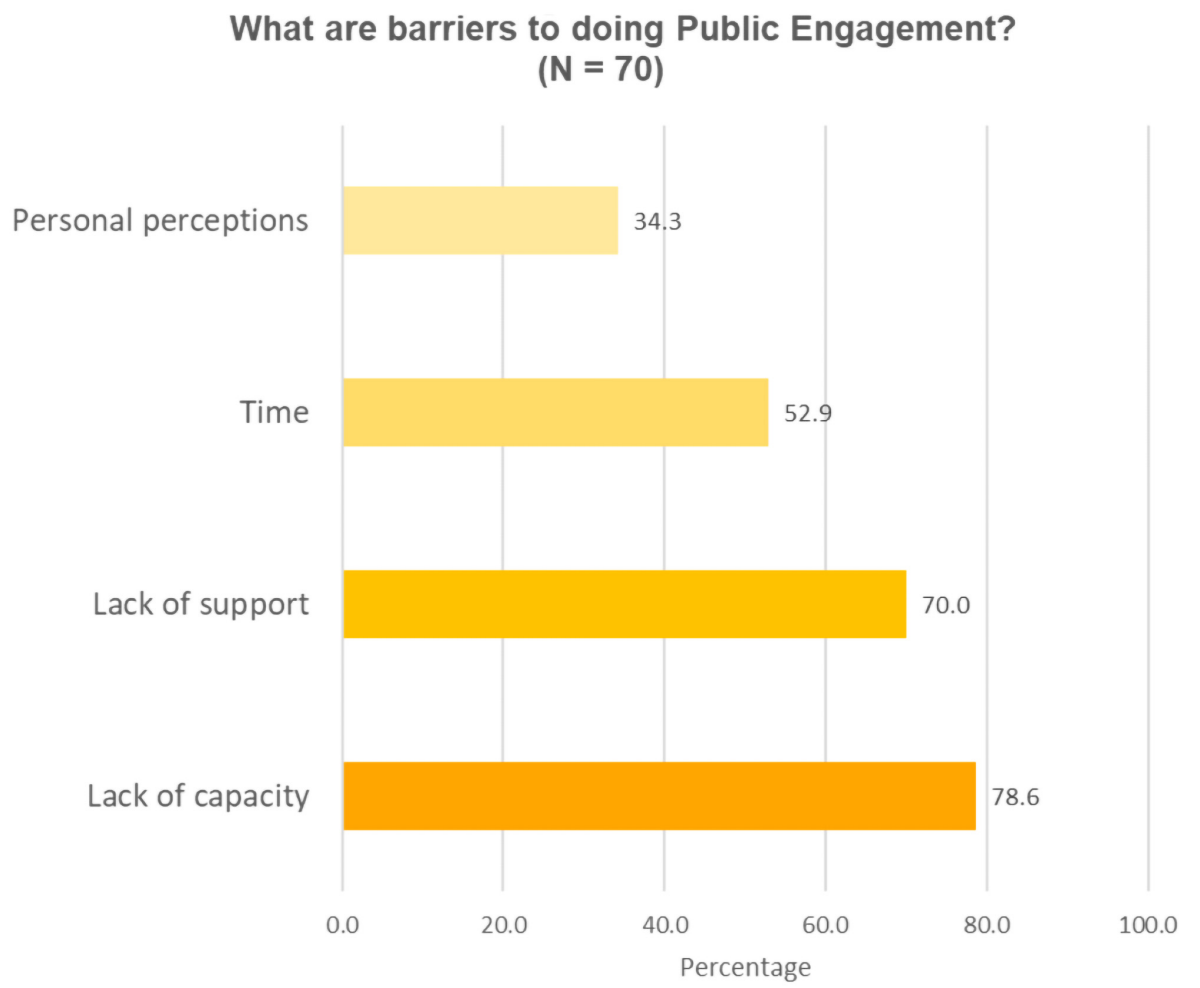
Perceived barriers to doing public engagement (Question 4).

**Figure 2b.**
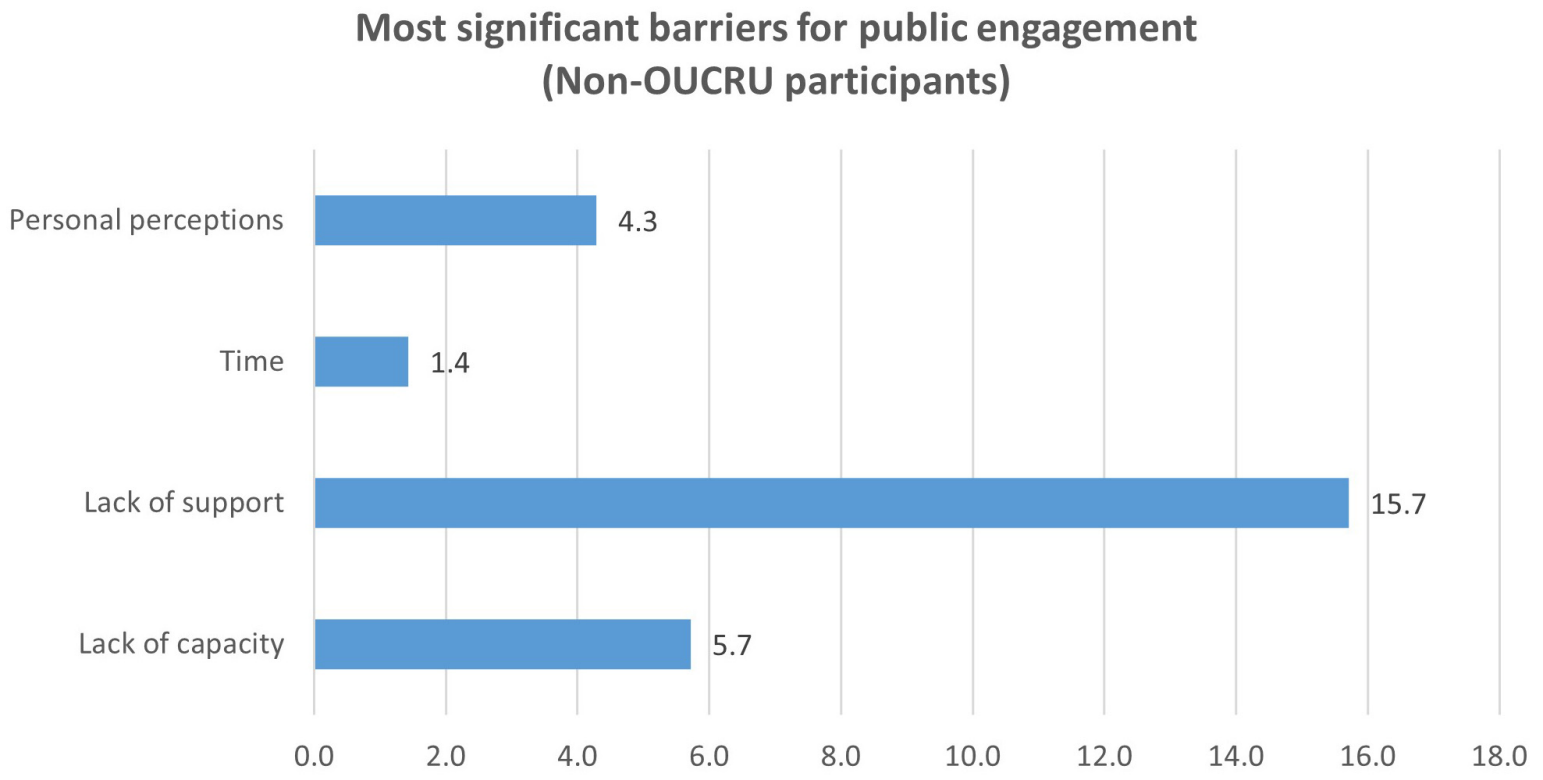
Perceived barriers to doing public engagement by participants from institutes outside OUCRU (Question 4).

**Table 6.** Participants’ needs in terms of public engagement capacity (Question 4).

LACK OF PUBLIC ENGAGEMENT CAPACITY (TOTAL 78.6%)				
**Skills, ideas and knowledge** ** of methods** to communicate science to various lay audiences	Using **appropriate** ** language** for various lay audiences	**Public** ** speaking** ** skill**	Lack of **understanding about** ** Public Engagement**	**Understanding ** **public ** **audience**
35.7%	20%	8.6%	7.1%	7.1%

**Table 7.** Participants’ perceived lack of support for public engagement (Question 4).

LACK OF PUBLIC ENGAGEMENT SUPPORT (TOTAL 70%)				
**Financial** and **human** **resources**	**Opportunities**, **platforms** and **network**	**Professional ** **recognition** of Public Engagement	**Policy** & **Culture**	**Training** opportunities
30%	20%	8.6%	7.1%	4.3%

**Table 8.** The barriers towards public engagement that originate from participants’ personal perceptions (Question 4).

PERSONAL PERCEPTIONS (TOTAL 34.3%)		
Lack of understanding of **audience's ** **interest/ needs/ background** ** knowledge**	The **complexity** of subject/ field	**Ability** and **preferences**
22.9%	7.1%	4.3%

Another barrier reported by researchers was a lack of support (49/70, 70%; Figure 2a). Researchers named a number of different issues which held them back from doing public engagement which collectively appears to be due to a lack of organizational mechanisms in research institutions ( Table 7). The two most commonly reported are the lack of funding and human resources, opportunities to get involved or network with people to collaborate on public engagement. The fact that public engagement is not recognised as part of researchers’ official responsibilities also reportedly contribute to the lack of support. A few researchers mentioned terms such as “policy” and “culture” in their responses as a barrier towards engagement. Some representative quotes include: “the possibility to speak the truth in this culture...”, “support from local authority, or national policy makers”. Related to this lack of support is the lack of time mentioned by over half the respondents (37/70, 52.9%; Figure 2a). This is not surprising given that public engagement is often not expected by institutions to be part of a researcher’s regular work. A third of participants (24/70, 34.3%; Figure 2a) reported barriers related to their own perceptions about the audience, their field of work and about themselves ( Table 8).

Analysing the data from non-OUCRU participants separately, the greatest barrier for them were lack of support (15.7%, 11/70; Figure 2b). This barrier was recognised by participants much more than the lack of capacity, as compared to OUCRU participants. For this group, lack of capacity and personal perception, specifically about public’s interest, needs, background knowledge, were similarly reported (5.7% and 4.3%, Figure 2b) and time constraint was a much less prominent barrier (4.3%, 3/70, Figure 2b).

***b) Participants confidence for communicating science with public***

**Figure 3.**
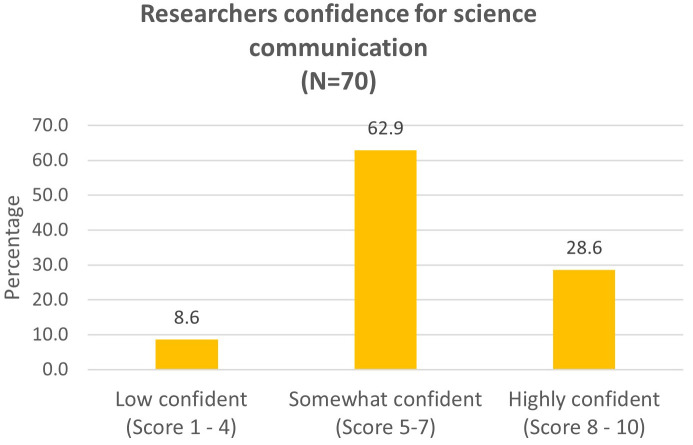
Researchers reported their confidence for communicating science to the public (Question 5a).

The participants rated their level of confidence in communicating about science to the public from 1 to 10 (Question 5, Figure 3). We classify a score of 8–10 as highly confident, 5–7 as somewhat confident and 1–4 as with low confidence. 71.4% of the participants had confidence score from 7 and below. Only 28.6% of the participants felt highly confident (score 8–10).

**Figure 4.**
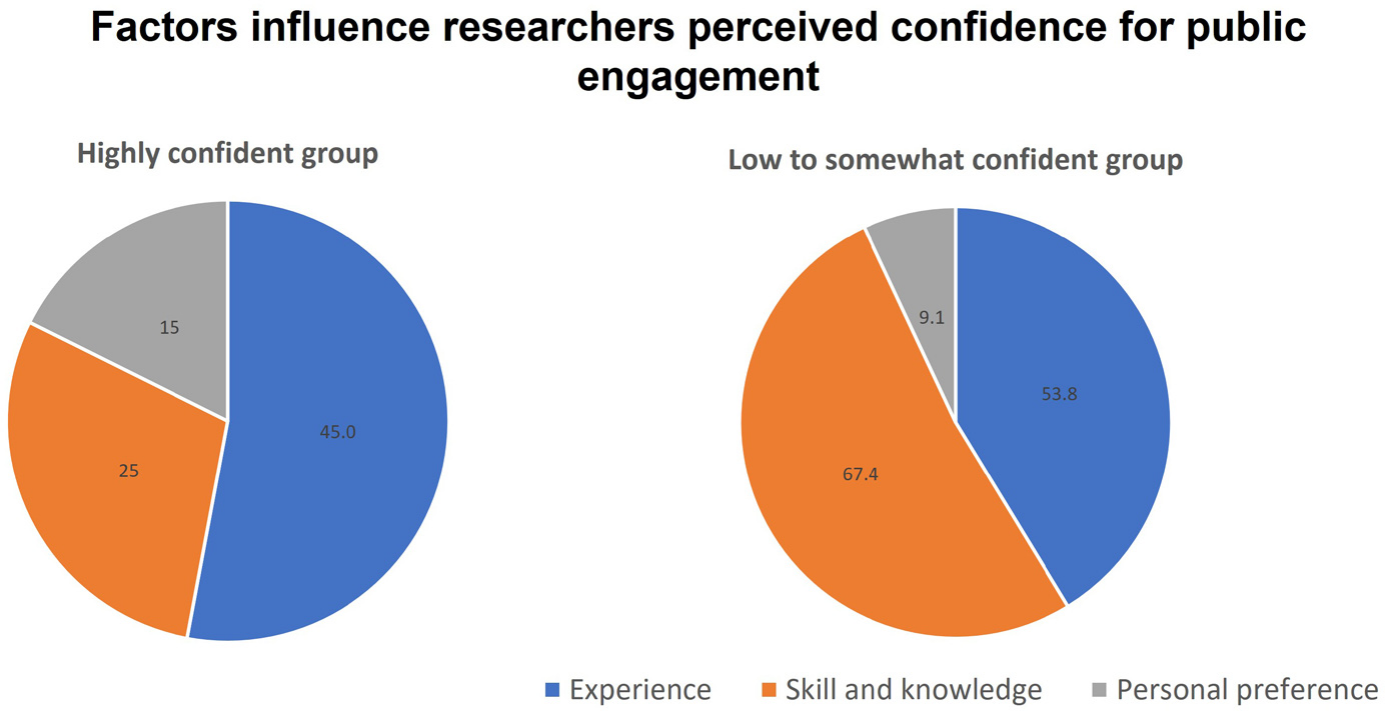
Factors that influence the confidence level of participants for communicating science with the public (Question 5b).

**Figure 5.**
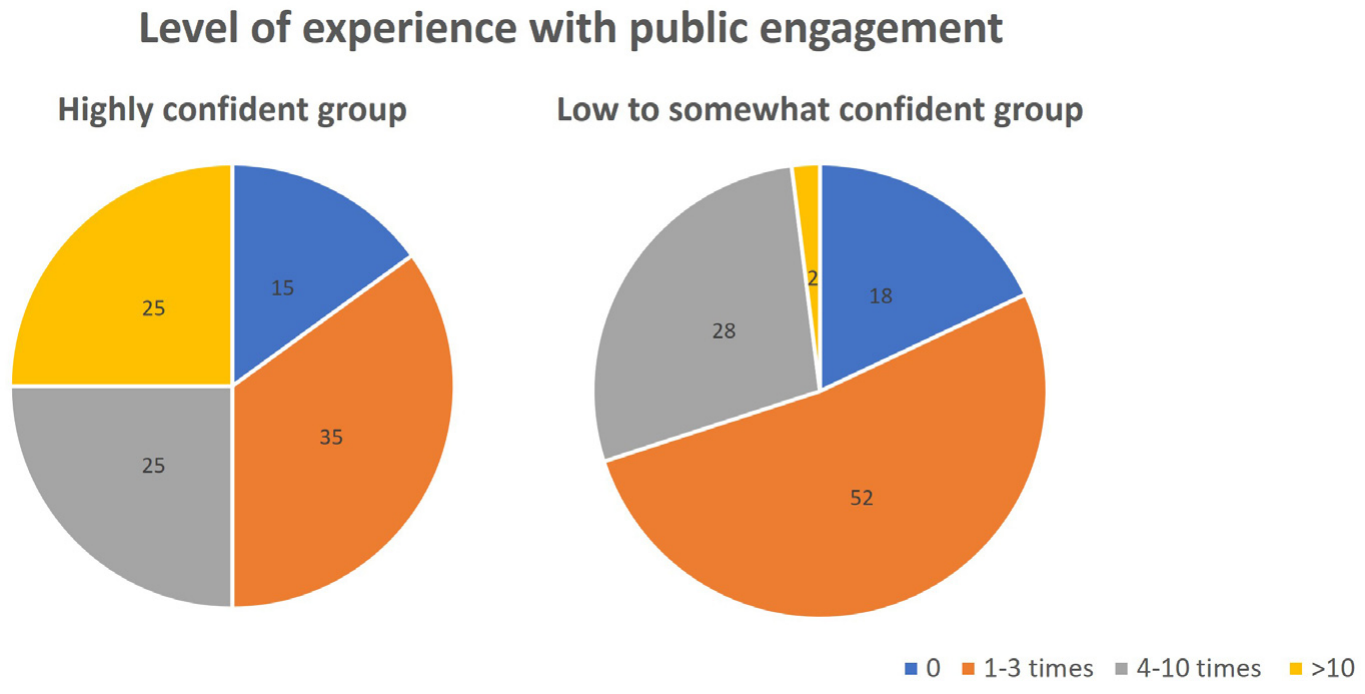
Participants level of experience with public engagement (times participating in public engagement) reflected against their confidence (Question 5b and 6b).

Participants were asked to explain their rating of confidence level and we were able to group the responses into 3 main factors affecting confidence: Experience, Skills and Knowledge, and Personal preference ( Figure 4). For participants who rated their confidence to be low and somewhat (score 1–7), they attributed the lack of skills and knowledge, and the lack of experience as almost equally important factors. On the other hand, highly confident researchers (score 8–10) were confident mainly because of their experience, while fewer of them attributed their confidence to skill and knowledge in their response. This finding is supported when reflecting the level of participants’ confidence with the number of times they participate in public engagement activities ( Figure 5). Most of the low to somewhat confident group is made up of people who participated 0–3 times in public engagement activities. In contrast, the high confidence group has more experienced participants (4–10 times and >10 times). Table 9 shows the specific ways researchers suggested to strengthen their science communication skills with the public.

**Table 9.** Suggestions for strengthening researchers science communication skills with the public (Question 7).

Suggestions to strengthen science communication skills	Percentage of participants (N=70)
Science communication skill workshops (science writing, science presentation…)	71.4
Training on developing participatory science activities for the public	61.4
Collaboration and networking with relevant individuals (researchers, public engagement practitioners) for public engagement projects	65.7
Training on social media and digital media for communicating science	52.9
Offering various platforms of science communication to gain exposure to the public audience	21.5

A less prominent factor influencing researchers’ confidence, but not to be dismissed, is how they personally perceive their own preference and ability ( Figure 4). Some researchers may find themselves naturally gravitating towards communicating and interacting activities with the public, others feel less natural joining such activities. Most of the responses are related to a concern about public speaking and face-to-face interaction with the public ( Table 10).

**Table 10.** Participants personal perceptions that contribute to their level of confidence for science communication with the public (Question 5b).

**Highly confident**	I enjoy being playful and having my audience challenge me.
	1. I have a need to communicate my science to the public. 2. I have experience with science communication with the public
	1. My current level of knowledge allows me to communicate science for an appropriate group of public audience. 2. I have skill for planning, presenting, explaining and good social skills. 3. I have an interest for doing work related to community.
**Somewhat ** **confident**	My hesitance with public speaking negatively affects my speech, thus affecting the impact of my communication between me and the public
	I am better in writing compared to speaking to public.
**Low confidence**	I am afraid of speaking in front of a crowd.
	I just don’t like activities that require interacting with non-science audiences.

## Discussion

With this survey, we have gained better understanding about researcher perceptions and attitudes towards engaging the public with science. We have collected evidence of common barriers researchers experienced and their needs for support in doing public engagement. This is particularly meaningful, given that current research on scientists’ perceptions, motivations and barriers for public engagement are mostly from Global North countries.

In our study most participants supported public engagement as a practice in science and research. It’s worth noting here that as a majority of the participants are affiliated with OUCRU which has an active public engagement department, the concept of public engagement might be familiar to many of them – influencing them to have a more positive predisposition towards the practice. Despite this, most participants didn’t recognise that public engagement is the two-way process generating mutual benefits of public engagement. Public engagement was commonly perceived as improving public literacy and understanding of science, rather than public participation in science and research. This is in line with earlier research showing that public engagement is still dominated by a deficit-style approach ( Weingart & Joubert, 2019). Interestingly, however, most participants appreciated the benefits of gaining public perspectives for their research, listing more benefits for their research than for the public and themselves personally. These findings imply that our researchers understand the mutual benefits in public engagement but lack the capacity to apply this mind set in practice. This highlights the need to continue emphasizing the role the public could have in participating in research, and also to provide researchers with training and experience to effectively engage in mutually beneficial dialogue with public audiences.

In 2015 the PCE group conducted a similar internal study including a survey and interviews (unpublished data). At this point, researchers did not identify much benefit for research when interviewed for their opinion on the importance of public engagement. Common answers among interviewees can be grouped in two main themes: (1) to make the public appreciate how research impacts their lives and (2) for the public to know useful science knowledge. Only one interviewee (from 27 interviews), mentioned that public engagement is for learning and understanding their research participants. Thus, our study in 2019 demonstrates a shift in thinking and how researchers now recognise the benefits of public engagement as not only for the public, but also for their research.

In recognition of the importance of researcher-led engagement the OUCRU PCE department has actively worked to bring communities and scientists together in our engagement activities and projects. Our researchers are supported to engage in dialogues with the local communities involved in their research to improve their understanding about community motivations and perceptions towards research. Some examples of this kind of engagement include the community advisory boards for research on the Hepatitis-C, in which members from the public provided their opinions and suggestions for some aspects of research. Our researchers are also invited to be involved in initiatives that make science accessible to the public in an engaging and entertaining way, such as through science theatre productions, debates and science cafés. We also support researchers-led public engagement initiatives by providing them with a funding scheme called Seed Awards. This survey is our attempt to better understand what the major barriers for researchers are to get involved in or implement public engagement in their research and thereby further strengthen our researcher support.

In our study, we identified similar barriers and needs for support to conduct public engagement as compared to the internal study in 2015. Lack of capacity was identified as the major barrier towards public engagement. Researchers indicated they need training to gain knowledge and skills to deliver science engagingly and effectively, to have ideas for science communication and understanding about public engagement. Capturing the areas of capacity researchers feel in need has helped orient the development of a training program to support the researchers. Communication training has been reported to effectively strengthen scientists’ self-perceptions of their interpersonal communication skills ( Besley *et al.*, 2015) as scientists typically perceived themselves and their colleagues as unskilful communicators ( Ecklund *et al.*, 2012). As seen from our survey, participants perceived themselves to have somewhat to low confidence for public engagement. In addition to knowledge and skills, the number of times researchers participate in public engagement is a major factor that influences researchers’ perceived confidence. Thus, in addition to providing trainings for building capacity, we emphasise the need to create opportunities for practicing communicating science with the public for researchers to gain experience, subsequently boosting their confidence. Moreover, with rich experience interacting with the public, their understanding of the public audience would be improved, which is thought to contribute to public engagement capacity according to our survey. 

Combined with the lack of capacity, participant’s ability and enthusiasm to engage with public audiences were limited by the lack of support and time constraints. This is in line with earlier research showing that scientists feel unsupported when it comes to public engagement ( Cerrato *et al*., 2018; Ho *et al*., 2020; Iqbal & Kar, 2022; Riley *et al.*, 2022; Rose *et al.*, 2020; Savage, 2013). The lack of support even led one of the participants in our survey to take a neutral stand towards the need to engage science with the public. Participants highlighted the lack of support in terms of funding, resources, opportunities, training and professional recognition. Some of these findings again highlight the need for development of training and practical opportunities for researchers to gain experience. Importantly, trainings on public engagement should be integrated into postgraduate curricular wherever possible to reduce the time constraints that might prevent researchers from joining the trainings. In addition to the need for trainings, researchers indicated the need to have public engagement personnel who can design and deliver public engagement programmes with and alongside the researchers. In term of funding, we have been providing Seed Awards for OUCRU researchers to pursue their public engagement initiatives. Furthermore, public engagement initiatives should be integrated in research proposals that rely on research uptake, community or public participation as that would encourage and enable researchers to allocate sufficient resources and time to carry out public engagement activities. This would also ensure that researchers see integrated public engagement activities as supporting research goals, instead of being a distraction. Although the national culture may be less conducive to public participation or researcher engagement, we suggest that research institutes can take responsibility for their internal environment. A participatory culture within research institutes needs to be established to provide an enabling environment for researchers to do public engagement. Such a culture could be fostered by recognising and publicising the public engagement work done by research staff through peer-review journal publications, internal communications and conferences for researchers involved in public engagement initiatives to network and share good practices.

Interestingly, the lack of support was even more prominent for participants from institutions outside OUCRU who reported it as the most common barrier to doing public engagement. The lack of capacity was much less recognised by this group as compared to OUCRU participants ( Figure 2a, Figure 2b). This is understandable as these non-OUCRU participants had already been actively involved in public engagement opportunities and thus perceived themselves to be capable due to their experience. Thus, for researchers from institutions outside OUCRU, the lack of organization support to do public engagement is a major barrier. 

Throughout our survey, personal perceptions emerged as a barrier for participants to do public engagement. Scientists’ self-efficacy for public engagement predicts their participation in such activities ( Besley, 2015; Besley *et al.*, 2013; Chapman *et al.*, 2014; Poliakoff & Webb, 2007). Indeed, despite the majority of participants in our study agreeing that public engagement is necessary, a few held neutral attitudes because of such personal perceptions. Personal perceptions are also a factor influencing participants’ confidence for public engagement. These perceptions were mostly about the public and participants own preference and ability for communicating science to the public. Thus, researchers need to have opportunities where they can get exposure and have dialogues with the public to challenge their perceptions about the public, better understand the public’s interest and needs, in turn removing these ‘mental blocks’ for them. Moreover, public engagement trainings need to present researchers with different perspectives and a range of science communication work, in comparison with their own perceptions. For instance, a portion of the participants associated public engagement capacity with having public speaking skills and some participants were concerned about face-to-face interaction with the public. Thus, there needs to be various formats of science communication available, suited for different researchers' preference and ability, so they can choose the method that they feel most comfortable with to share their science. Based on the survey findings, we have developed recommendations for further supporting researchers with public engagement. A summary of our recommendations is presented in Table 11. 

**Table 11.** Summary of recommendations for supporting researchers with public engagement.

Recommendations		Expected outcomes
**Training**	• Emphasise the role and benefits of public participation in research. • Provide skills to engage effectively in mutually beneficial dialogue with public audiences. • Regularly integrate public engagement trainings into postgraduate curricular. • Show different perspectives and range of science communication work.	• Increase knowledge and skills related to public engagement. • Strengthen scientists’ self-perceptions and remove mental blocks towards public engagement.
**Practice**	• Enable experience to engage effectively in mutually beneficial dialogue with public audiences. • Develop and offer regular opportunities in diverse formats for communicating science with the public. • Continue our Seed Awards Scheme.	• Better understand the public audience. • Verify personal perceptions that are mental blocks towards engagement. • Build experience and confidence for doing public engagement. • Researchers can choose the form of science communication that is most suitable to their preference and ability. • Improve participation and attitude towards public engagement.
**Professional** ** recognition**	• Annual conferences or meetings • Publication of researcher-led engagement (Peer- review journals) • Recognition in internal communications (e.g., Institutional newsletter)	• Sharing good practice and ideas. • Networking with like-minded peers interested in public engagement. • Raising awareness about the role of public engagement for science and research. • Foster a culture of public engagement in research.
**Consultation**	• Consultant group - Public engagement personnel who can design and deliver public engagement programmes with and alongside the researchers.	• Professionally support researchers with public engagement.

Our survey has revealed perspectives of a group of researchers in Vietnam, Nepal and Indonesia on public engagement. We acknowledge that the predominance of respondents from OUCRU programme sites lends a degree of bias and that our results may not accurately represent the situation in other institutes in Southeast Asia. OUCRU has an active public engagement department that promotes researcher participation in engagement initiatives and therefore, the OUCRU participants have had a high level of exposure towards public engagement. Additionally, the unresponsiveness of researchers from other institutions to the survey invitation may indicate an underlying lack of awareness or interest in the practice of public engagement in Vietnam, Nepal and Indonesia. Another source of bias may come from the fact that participants who were already interested in public engagement may be more likely to respond to the survey than those who were not so interested. It would be important for future studies to investigate the perceptions of those who are not (yet) interested in public engagement and to explore the reasons researchers might avoid or oppose public engagement.

## Conclusion

While the participants in our survey largely see public engagement as a necessary practice, they experienced various barriers to take part in public engagement. Our study reported barriers similar to those in previous studies, which include the lack of time, skills and an enabling environment to support public engagement. Unlike the studies that reported time constraint as the greatest barrier ( Ho *et al*., 2020; Iqbal & Kar, 2022; Woitowich *et al*., 2022), respondents in our study percieved, time as a smaller barrier compared to the lack of skills. In contrast to our finding, the lack of skill was not at all reported as a barrier in a study by Woitowich *et al*., 2022. We interpret this difference resulting from the fact that in situations where public engagement is more commonly practiced and supported, lack of time is a greater barrier than capacity. Additionally, our study also identified researchers’ personal perceptions about their preference, ability and the practice of public engagement as a barrier, which had been reported occasionally ( Ho *et al*., 2020). These findings from our study indicate that there remains a great need to support researchers to better understand the nature of public engagement and to acquire core competencies to engage the public with science. There has been some level of support for public engagement at our institution, however it is evident that more is needed to build researchers capacity for public engagement and build an enabling institutional environment that gives researchers professional recognition for their engagement work. Moving forward, the findings from the survey are informing our development of training and activities that will support researchers with public engagement.

## Data Availability

Zenodo: A study on biomedical researchers' perspectives on public engagement in Southeast Asia (Version 3. https://doi.org/10.5281/zenodo.7870369 ( Han & Chambers, 2023). This project contains the following underlying data: Researchers perceptions of PCE_raw data_v2.xlsx Data are available under the terms of the Creative Commons Attribution 4.0 International license (CC-BY 4.0).

## References

[ref-1] Besley JC : What do scientists think about the public and does it matter to their online engagement? *Sci Public Policy.* 2015;42(2):201–214. 10.1093/scipol/scu042

[ref-2] Besley JC Dudo A Storksdieck M : Scientists’ views about communication training. *J Res Sci Teach.* 2015;52(2):199–220. 10.1002/tea.21186

[ref-3] Besley JC Oh SH Nisbet M : Predicting scientists' participation in public life. *Public Underst Sci.* 2013;22(8):971–987. 10.1177/0963662512459315 23825262

[ref-4] Bodmer WF : The public understanding of science. Report of a Royal Society ad hoc group endorsed by the council of the Royal Society. The Royal Society,1985. Reference Source

[ref-5] Cerrato S Daelli V Pertot H : The public-engaged scientists: Motivations, enablers and barriers. *Research for All.* 2018;2(2):313–322. 10.18546/RFA.02.2.09

[ref-6] Chapman S Haynes A Derrick G : Reaching "an audience that you would never dream of speaking to": influential public health researchers' views on the role of news media in influencing policy and public understanding. *J Health Commun.* 2014;19(2):260–273. 10.1080/10810730.2013.811327 24156565

[ref-7] Ecklund EH James SA Lincoln AE : How academic biologists and physicists view science outreach. *PLoS One.* 2012;7(5): e36240. 10.1371/journal.pone.0036240 22590526 PMC3348938

[ref-8] Factors affecting public engagement by UK researchers. *Wellcome.* 2016; (Accessed: January 13, 2023). Reference Source

[ref-9] Han TDT Chambers M : A study on biomedical researchers' perspectives on public engagement in Southeast Asia (Version 3). Zenodo. [Data set],2023. 10.5281/zenodo.7870369 PMC10521067 37766854

[ref-10] Ho SS Looi J Goh TJ : Scientists as public communicators: individual- and institutional-level motivations and barriers for public communication in Singapore. *Asian J Commun.* 2020;30(2):155–178. 10.1080/01292986.2020.1748072

[ref-11] Hofman P : Participation in Southeast Asian Pollution Control Policies. *Participation and the Quality of Environmental Decision Making.* 1998;14:287–305. 10.1007/978-94-011-5330-0_21

[ref-12] Iqbal S Kar B : A survey to gather perspectives of DBT/Wellcome Trust India Alliance-funded researchers on public engagement with science [version 2; peer review: 2 approved]. *Wellcome Open Res.* 2022;6:269. 10.12688/wellcomeopenres.17120.2 35509370 PMC9021671

[ref-14] Leshner AI : Public Engagement with Science. *Science.* 2003;299(5609):977. 10.1126/science.299.5609.977 12586907

[ref-15] Lin Heng L : Public Participation in the Environment: A South-East Asian Perspective. In: Donald M. Zillman, Alastair Lucas, and George (Rock) Pring (eds), *Human Rights in Natural Resource Development: Public Participation in the Sustainable Development of Mining and Energy Resources. * 2002;650–677. 10.1093/acprof:oso/9780199253784.003.0018

[ref-100] Lo YY Peters HP : Taiwanese life scientists less “medialized” than their Western colleagues. * Public Underst Sci.* 2015;24(1):6–22. 10.1177/0963662513513863 24368762

[ref-26] Luck A : International public engagement Gathering views of international public and community engagement with research across Africa and India. *Wellcome*.2016; (Accessed: 06 September 2023). Reference Source

[ref-16] Mayor F : Science for the 21st century: A new commitment.In: A. M. Cetto, S. Schneegans, & H. Moore (Eds.), *Proceedings of the World Conference on Science. *UNESCO; Banson,1999;26–28. Reference Source

[ref-17] National Coordinating Centre for Public Engagement, NCCPE. Reference Source

[ref-18] Pew Research Center: Americans Value U.S. Role as Scientific Leader, but 38% Say Country Is Losing Ground Globally. 2022. Reference Source

[ref-19] Poliakoff E Webb TL : What Factors Predict Scientists’ Intentions to Participate in Public Engagement of Science Activities? *Sci Commun.* 2007;29(2):242–263. 10.1177/1075547007308009

[ref-20] Public attitudes to science 2011. ipsos, (no date), (Accessed: January 13,2023). Reference Source

[ref-13] Riley J Joubert M Guenther L : Motivations and barriers for young scientists to engage with society: perspectives from South Africa. *Int J Sci Educ B.* 2022;12(2):157–173. 10.1080/21548455.2022.2049392

[ref-21] Rose KM Markowitz EM Brossard D : Scientists' incentives and attitudes toward public communication. *Proc Natl Acad Sci U S A.* 2020;117(3):1274–1276. 10.1073/pnas.1916740117 31911470 PMC6985784

[ref-22] Savage L : A View from the Foothills: Public Engagement among Early Career Researchers. *Political Stud Rev.* 2013;11(2):190–199. 10.1111/1478-9302.12010

[ref-23] Weingart P Joubert M : The conflation of motives of science communication - causes, consequences, remedies. *J Sci Commun.* 2019;18(03). 10.22323/2.18030401

[ref-24] Wellcome Global Monitor. 2018; (Accessed: February 9, 2023). Reference Source

[ref-25] Woitowich NC Hunt GC Muhammad LN : Assessing motivations and barriers to science outreach within academic science research settings: A mixed-methods survey. *Front Commun.* 2022;7: 907762. 10.3389/fcomm.2022.907762

