# Supplementary material for: A study on biomedical researchers’ perspectives on public engagement in Southeast Asia
Source: Wellcome Open Res. 2023 Sep 8;8:202. Originally published 2023 May 10. [Version 2] doi: 10.12688/wellcomeopenres.19040.2 (PMC10521067; doi:10.12688/wellcomeopenres.19040.2)
Supplement: Peer review history

# Reviewer report: Reviewer response for version 2

**DOI:** 10.21956/wellcomeopenres.21866.r66783

**Published:** 2023-09-12

**Authors:** Marina Joubert

**Affiliations:** 1 Centre for Research on Evaluation, Science and Technology (CREST), Stellenbosch University, Stellenbosch, South Africa

I hereby confirm that I’m satisfied that the authors have addressed my previous concerns sufficiently, and that the article is now approved (from my perspective).

Is the work clearly and accurately presented and does it cite the current literature?

Yes

If applicable, is the statistical analysis and its interpretation appropriate?

Yes

Are all the source data underlying the results available to ensure full reproducibility?

Yes

Is the study design appropriate and is the work technically sound?

Yes

Are the conclusions drawn adequately supported by the results?

Yes

Are sufficient details of methods and analysis provided to allow replication by others?

Yes

Reviewer Expertise:

Science communication; Public engagement with science

I confirm that I have read this submission and believe that I have an appropriate level of expertise to confirm that it is of an acceptable scientific standard.

# Reviewer report: Reviewer response for version 2

**DOI:** 10.21956/wellcomeopenres.21866.r66784

**Published:** 2023-09-12

**Authors:** Joanna Sleigh

**Affiliations:** 1 Swiss Federal Institute of Technology (ETH Zurich), Zurich, Switzerland

No further comments.

Is the work clearly and accurately presented and does it cite the current literature?

Yes

If applicable, is the statistical analysis and its interpretation appropriate?

Yes

Are all the source data underlying the results available to ensure full reproducibility?

Yes

Is the study design appropriate and is the work technically sound?

Yes

Are the conclusions drawn adequately supported by the results?

Yes

Are sufficient details of methods and analysis provided to allow replication by others?

Partly

Reviewer Expertise:

Bioethics

I confirm that I have read this submission and believe that I have an appropriate level of expertise to confirm that it is of an acceptable scientific standard.

# Reviewer report: Reviewer response for version 1

**DOI:** 10.21956/wellcomeopenres.21109.r58608

**Published:** 2023-06-08

**Authors:** Joanna Sleigh

**Affiliations:** 1 Swiss Federal Institute of Technology (ETH Zurich), Zurich, Switzerland

This study examines the perspectives and needs of local researchers regarding public engagement in the context of Southeast Asia, focusing on the Oxford University Clinical Research Unit (OUCRU) in Vietnam, Nepal, and Indonesia. The study employed an online survey with both open-ended and closed-ended questions, gathering responses from 70 researchers. The study design appears appropriate for the research objectives, however I recommend adding more details about the method improve this manuscript.

 Specifically, while the methods and analysis are described, some more details would enhance the quality of reporting. For example, the survey tool used (Google Forms) is mentioned, but specifics as to how the survey questions were developed is not provided. Were the multiple choice questions informed by any literature? Were these questions piloted or pre-tested? How was the test validity and reliability assessed? It would also help to have an Expansion on the limitations of the sample and method in the discussion (opposed to conclusion).

Is the work clearly and accurately presented and does it cite the current literature?

Yes

If applicable, is the statistical analysis and its interpretation appropriate?

Yes

Are all the source data underlying the results available to ensure full reproducibility?

Yes

Is the study design appropriate and is the work technically sound?

Yes

Are the conclusions drawn adequately supported by the results?

Yes

Are sufficient details of methods and analysis provided to allow replication by others?

Partly

Reviewer Expertise:

Bioethics

I confirm that I have read this submission and believe that I have an appropriate level of expertise to confirm that it is of an acceptable scientific standard, however I have significant reservations, as outlined above.

# Reviewer report: Reviewer response for version 1

**DOI:** 10.21956/wellcomeopenres.21109.r57245

**Published:** 2023-05-26

**Authors:** Marina Joubert

**Affiliations:** 1 Centre for Research on Evaluation, Science and Technology (CREST), Stellenbosch University, Stellenbosch, South Africa

This study from Southeast Asia adds new knowledge and insights regarding researchers’ perspectives on public engagement from a part of the world that has been less well studied in this regard. The authors acknowledge that it is a modest study in terms of its scope and that most respondents are from inside the Oxford University Clinical Research Unit (OUCRU) where there is a dedicated public engagement team. Despite this, the study presents interesting and noteworthy results.

 In terms of relevant earlier studies, I recommend that the authors look at the work by YY Lo from Taiwan (see, for example, Lo, Y.-Y. & Peters, H.P. 2015. Taiwanese life scientists less “medialized” than their Western colleagues. Public Understanding of Science, 24(1):6–22.) ^1^

 Data collection: Please change ‘approximately’ 273 people on the email list … it is a precise number? You have to know exactly how many people were emailed. It is not clear to me how many people were emailed (or asked to complete the survey), and therefore it is not clear what response rate you had when 70 researchers responded. Please clarify as close as possible how many people were emailed (at the time it was sent out) and what response rate you achieved. [Also check in this paragraph – there should be a comma after the word ‘group’ (not a full stop).]

 You say that two coders used the same coding scheme to analyse the data and that there were minimal differences. It is better (something to keep in mind for future work) to perform a proper inter-rater reliability test.

 Ethics: I am surprised that you mention that ethical approval was not required, especially since not all of those approached to take part in the study were in OUCRO. It is unusual (in my experience) to claim that “participation and distribution by representatives from the 14 external research institutes was a demonstration of institutional approval and consent”. Once again, this is not something you can change now, but perhaps something to reconsider in future work of this kind.

 Results: You have to provide some information about the response rate … what percentage do 70 people represent? It is also important to reflect on the response rate, given that this survey mostly targeted researchers in an institutional environment where there is a focus on public engagement.

 The rest of the results are presented clearly.

 In your discussion, you compare your findings with those of an earlier internal study in 2015, and you also compare with findings from studies elsewhere in the world. I would have liked to see some conclusions about what you thought were different (or not) for researchers in Southeast Asia. In other words, are the motivations and barriers for public engagement the same everywhere, or do you think there are notable differences in your specific region and context?

Is the work clearly and accurately presented and does it cite the current literature?

Yes

If applicable, is the statistical analysis and its interpretation appropriate?

Yes

Are all the source data underlying the results available to ensure full reproducibility?

Yes

Is the study design appropriate and is the work technically sound?

Yes

Are the conclusions drawn adequately supported by the results?

Yes

Are sufficient details of methods and analysis provided to allow replication by others?

Yes

Reviewer Expertise:

Science communication; Public engagement with science

I confirm that I have read this submission and believe that I have an appropriate level of expertise to confirm that it is of an acceptable scientific standard, however I have significant reservations, as outlined above.

## References

[1] : Taiwanese life scientists less "medialized" than their Western colleagues. *Public Underst Sci* .2015;24(1) : 10.1177/0963662513513863 6-22 10.1177/0963662513513863 24368762
